# Awareness, Attitudes, and Barriers Toward Artificial Intelligence in Dentistry: A Cross-Sectional Survey of Dental Professionals in Kuwait

**DOI:** 10.3390/dj14100618

**Published:** 2026-09-24

**Authors:** Kawther Ali, Alghalyah Al-Ali, Maryam Safar, Muawia Qudeimat

**Affiliations:** 1 Department of Restorative Sciences, College of Dentistry, Kuwait University, Sabah Al Salem University City, Shadadiya, Safat 13060, Kuwait; 2 Ministry of Health, Sulaibikhat, Safat 13001, Kuwait; alghalyahjm@gmail.com (A.A.-A.); ms.maryam@hotmail.com (M.S.); 3 Department of Developmental Sciences, College of Dentistry, Kuwait University, Sabah Al Salem University City, Shadadiya, Safat 13060, Kuwait

**Keywords:** artificial intelligence, dentistry, dental professionals, awareness, attitudes, barriers, digital dentistry, Kuwait, cross-sectional survey

## Abstract

**Background/Objectives**: Artificial intelligence (AI) is rapidly entering dental education and clinical practice, yet data on how dental professionals in the Middle East perceive it are scarce. This study assessed the awareness, attitudes, and perceived barriers toward AI among dental professionals in Kuwait, and compared responses across specialty, work sector, place of training, and experience, in order to inform regional education and adoption strategies. **Methods**: A cross-sectional online survey of 27 items across five domains (demographics, knowledge and awareness, clinical practice, attitudes, and barriers and concerns) was distributed to dental care providers practicing in Kuwait’s government, private, and academic sectors between January 2024 and January 2025. An investigator-defined composite “belief” score (range 0–9), calculated as the sum of affirmative responses to nine attitude and expectation items (Kuder–Richardson 20 = 0.79), was categorized a priori as low (0–4), moderate (5–7), or high (8–9). This score was constructed for the present study to summarize overall positive orientation toward AI and is not a previously validated instrument. Categorical data were compared using the chi-square or Fisher exact test, and ordinal logistic regression was used to identify predictors of a higher belief-score category. **Results**: A total of 321 dental professionals accessed the survey, and 300 with complete belief-score data formed the analytic sample. Just over half were aware of AI tools designed for dentistry (56.3%), yet exposure was limited: only 38.7% had attended an AI-related lecture, workshop, or webinar, 29.0% had read AI research, and 15.7% had used AI-assisted tools, while 94.0% were interested in AI training and 92.0% were open to adopting AI if beneficial. Among the 292 respondents who answered the multiple-response barrier item, the most frequently reported barriers were a lack of training and expertise (71.2%), technical challenges (63.4%), and cost (56.5%); these did not differ significantly across work sector or specialty. High composite beliefs were more common among male than female respondents (44.1% vs. 25.3%, *p* = 0.003), and in ordinal regression male gender (estimate = 0.736, *p* = 0.004), general dentistry (estimate = 0.924, *p* = 0.003), and prosthodontics (estimate = 1.120, *p* = 0.003) independently predicted higher beliefs (model χ^2^ = 25.56, df = 9, *p* = 0.002). **Conclusions**: Dental professionals in Kuwait show high enthusiasm for AI but limited training and hands-on exposure, and a lack of training and expertise was the leading perceived barrier (71.2% of the 292 respondents answering this item) across all sectors and specialties. Targeted, hands-on AI training and the integration of AI content into dental curricula are needed to support effective and responsible adoption in Kuwait.

## 1. Introduction

Advances in technology have enhanced many aspects of modern life, and healthcare is among the fields that have benefited most. One such advance is artificial intelligence (AI). Dentistry is undergoing a significant transformation as AI is integrated into both education and clinical practice, with applications that increasingly include diagnostic assistance and support for treatment planning across implantology, prosthodontics, endodontics, and other specialties.

AI has the potential to enhance caries detection on radiographs [1]. In prosthodontics, AI offers benefits for both removable and fixed prostheses: by automating margin detection after tooth-preparation scanning, it can save time and contribute to more precise crown design with an ideal fit and occlusion [2]. Its predictive capabilities also extend to estimating the longevity and failure rates of crowns, aiding treatment planning and outcome prediction. In removable prosthodontics, AI assists in designing prostheses and in predicting their impact on facial appearance and phonetics [3]. In implantology, AI contributes to surgical planning by determining optimal implant positions, predicting angulation, and assessing the need for bone grafting [4]. In endodontics, AI is a valuable tool for the early detection of periapical lesions and vertical root fractures, for determining working length, and for previewing root-canal morphology, thereby reducing the risk of misdiagnosis and improving outcomes [5]. Collectively, these applications illustrate how AI may improve diagnosis, treatment, and outcomes in dentistry.

Several studies have examined AI awareness among health professionals. A systematic review by Dashti et al. [6] reported a basic AI knowledge score of 58.62% among dental students compared with 71.75% among dentists, although more students (72.01%) than dentists (62.60%) believed AI could improve dental practice. Parthasarathy et al. [7] found that most dentists believed in the potential benefits of AI; nevertheless, 50.0% were concerned about its ethical implications, while 70.3% were willing to adopt AI in practice. A scoping review by Aldakhil et al. [8] reported that, on average, 44.13% of dentists felt threatened by AI or felt it might replace them. Across this literature, awareness typically clusters around half of respondents, and the most frequently cited barriers are a lack of training and limited resources [8].

Although AI awareness, knowledge, and attitudes have been explored widely, data from the Middle East remain limited. To our knowledge, no data from Kuwait have been published, and few studies analyze responses by professional subgroup. This study addresses that gap. To our knowledge, it is the first to investigate dental professionals’ awareness, attitudes, and barriers toward AI in Kuwait, and it does so while comparing responses across specialty (including specialists versus general dentists), work sector (government, private, and academic), place of undergraduate training, and years of experience. Because attitudes toward AI are likely to shift as the technology advances rapidly, contrasting Kuwaiti data with the existing literature is both timely and informative. The aim of this study was to investigate the awareness, attitudes, barriers, and applications of AI among dental professionals in Kuwait and to compare them across these subgroups. The null hypothesis was that there is no difference among dental professional subgroups in awareness, attitudes, barriers, or applications of AI.

## 2. Materials and Methods

### 2.1. Study Design and Participants

This cross-sectional survey was designed and distributed through the SurveyMonkey online platform (SurveyMonkey Inc., San Mateo, CA, USA). The study was approved by the Health Sciences Center Ethical Committee of Kuwait University (reference VDR/EC-515; approved 21 December 2023, following committee review on 20 December 2023). The target population comprised dental care providers practicing in Kuwait in the government, private, and academic sectors. Participants were recruited in their workplaces and through social media (WhatsApp version 2.26.36.74 (WhatsApp LLC, Meta Platforms, Inc.; Menlo Park, CA, USA)) and email. Inclusion criteria were dentists practicing in Kuwait who were aged 18 years or older. Exclusion criteria were individuals younger than 18 years and those not working in the dental field. Participants were informed that participation was voluntary and uncompensated. The survey remained open for 12 months (January 2024 to January 2025).

Because the survey link was disseminated through workplaces, social media, and email, the total number of dental professionals who received or viewed the invitation could not be determined, and a conventional response rate could therefore not be calculated. The survey was anonymous and no identifying information was collected; the platform was configured to allow only one response per device/browser to reduce the risk of duplicate participation. A post hoc screening of the exported dataset identified no duplicate platform-generated respondent identifiers and no identical records among the 300 respondents in the analytic sample. To reduce recruitment-related bias, the invitation was disseminated through several complementary channels (distribution in workplaces, WhatsApp, and email), was addressed to dentists in all three practice sectors irrespective of their familiarity with AI, and the survey was kept open for 12 months so that practitioners with less frequent access to these channels could participate. The consent statement presented the study as a survey of awareness of and attitudes toward AI, so that participation did not presuppose prior knowledge of or experience with AI.

### 2.2. Questionnaire

The questionnaire was anonymous and comprised 27 items across five domains—participant demographics, knowledge and awareness of AI, clinical practice applications of AI, attitudes toward AI, and barriers and concerns regarding AI (Table 1 and Table 2)—together with a participant consent statement. It contained multiple-choice and open-ended questions.

The questionnaire was developed by the research team in three stages. First, a review of the literature on dental professionals’ awareness of and attitudes toward AI was undertaken to define the domains of the instrument and to identify candidate items. The five domains were informed by the constructs synthesized in a systematic review of dentists’ and dental students’ attitudes, knowledge, and perceptions of AI [6], and the individual items were adapted from the questionnaires used in previously published primary surveys of dentists and dental students [9,10]. Second, the draft questionnaire was reviewed for content and face validity by four faculty members of Kuwait University with expertise in general, pediatric, prosthodontic, and orthodontic dentistry and in AI imaging. They assessed the relevance of each item to its intended domain and the clarity and comprehensiveness of the wording; items were reworded on the basis of their feedback. This review was qualitative, and no content validity index was calculated. No further quantitative psychometric evaluation (for example, test–retest reliability or factor analysis) was undertaken. The draft instrument was piloted with three participants before the survey link was distributed to the target population. The purpose of the pilot was refinement of the instrument rather than data collection, and it was not designed as a psychometric evaluation. Their responses were used solely to refine the questionnaire and were not included in the analytic dataset. The feedback was discussed by the research team and the questionnaire was revised accordingly. Specifically: the lists of undergraduate and postgraduate education countries were finalized; the response options for years of experience were finalized; the wording of several items was simplified to remove ambiguity; and items judged redundant were removed. The revised version was the instrument distributed to participants. The internal consistency and dimensionality of the nine items combined into the composite belief score were therefore examined post hoc in the study sample, as described in Section 2.3 and reported in Section 3.3.

### 2.3. Statistical Analysis

No formal a priori sample-size calculation was performed; all dental professionals who responded during the study period were included as a convenience sample. Owing to the exploratory nature of the study and the absence of prior Kuwaiti data on this topic, no reliable estimates were available to support an a priori sample-size calculation. Accordingly, all eligible respondents during the study period were included. A total of 321 individuals accessed the survey. Twenty-one respondents (6.5%) did not answer all nine items contributing to the composite belief score and were therefore excluded, leaving a primary analytic sample of 300 respondents; descriptive analyses of the awareness, clinical practice, attitude, and barrier domains are reported within this sample, with item-level valid-response denominators stated in each table where item non-response occurred. The multivariable ordinal regression was performed on the 289 respondents with complete data across the outcome and all model covariates; the 11 additional exclusions corresponded to respondents reporting a mixed or other work sector, which could not be assigned to one of the three main sector categories. Missing data were not imputed; all analyses were complete-case analyses, with item-level valid-response denominators reported in the tables. Non-response was scattered across items and showed no apparent systematic pattern. All analyses were performed using IBM SPSS Statistics (version 30; IBM Corp., Armonk, NY, USA) and Python (version 3.12; Python Software Foundation, Wilmington, DE, USA).

Terminology is applied consistently throughout the manuscript: “awareness” denotes reported familiarity with the existence of AI tools in dentistry; “knowledge” and “exposure” denote reported engagement with AI content, such as lectures, workshops, webinars, and peer-reviewed research; “attitudes” denote evaluative and expectational responses to AI; “barriers” denote obstacles to AI implementation reported by respondents; and “beliefs” refers exclusively to the investigator-defined composite score defined below, which aggregates nine attitude and expectation items. The term “perceptions” is used only when describing the findings of other studies that adopt it. A composite belief score (range 0–9) was derived by summing affirmative (“Yes”) responses to nine items reflecting positive attitudes, perceived benefits, and readiness to adopt AI in dentistry. The score was developed specifically for this exploratory study as a pragmatic indicator of overall positive orientation toward AI. Equal weighting was assigned to all items because no validated evidence was available to support differential weighting. The score was categorized into low (0–4), moderate (5–7), and high (8–9) belief groups using investigator-defined thresholds intended to facilitate interpretation and subgroup comparisons. The internal consistency of the nine dichotomously scored items was evaluated post hoc in the analytic sample using the Kuder–Richardson formula 20 (KR-20), the form of Cronbach’s alpha appropriate for dichotomous items, with a 95% confidence interval calculated by Feldt’s method; corrected item–total correlations, the mean inter-item correlation, and the value of KR-20 with each item deleted were also examined. Whether the nine items could reasonably be summed into a single score was assessed by principal component analysis of the inter-item correlation matrix (Pearson correlations), with the number of components to retain determined by parallel analysis against 1000 random datasets; the Kaiser–Meyer–Olkin (KMO) measure of sampling adequacy and Bartlett’s test of sphericity were used to confirm the suitability of the correlation matrix for this analysis. Categorical variables were compared using the chi-square test (expected cell count ≥ 5) or the Fisher exact test (expected cell count < 5). Predictors of belief category were examined using ordinal (proportional odds) logistic regression with a logit link, including gender, specialty, place of undergraduate and postgraduate education, years of experience, and work sector. The proportional-odds assumption was assessed using a test of parallel lines. Multicollinearity among predictor variables was evaluated using variance inflation factors (VIFs). Regression results are presented as adjusted odds ratios (ORs) with 95% confidence intervals (CIs). A two-sided *p*-value < 0.05 was considered statistically significant.

Place of undergraduate education was dichotomized into graduates of countries ranked among the global top 10 in artificial intelligence according to the 2024 Global AI Index versus graduates of all other countries. Among study participants, only four of these top-ranked countries were represented, namely the United States, United Kingdom, France, and India, and were therefore classified within the top-10 category [11]. Years of experience were dichotomized to facilitate interpretation and to distinguish earlier-career clinicians (1–6 years) from those with more established professional experience (>6 years), while maintaining adequate group sizes for the ordinal regression analysis. Specialty was grouped as general dentistry, orthodontics, prosthodontics, and others (periodontics, oral surgery, endodontics, pediatric dentistry, and oral medicine), because the numbers of respondents in the individual specialties within the “others” group were too small to support stable stratified estimates; the heterogeneity of this combined group is considered in the Discussion. Although date of birth was collected, age was not analyzed as a separate variable because it overlaps substantially with years of professional experience, which was retained as the more practice-relevant indicator.

## 3. Results

### 3.1. Participant Characteristics

The characteristics of the 300 respondents in the analytic sample are summarized in Table 3. Most were female (60.7%), and the largest specialty group was general dentistry (37.0%). Most respondents worked in the government sector (64.0%), and just over half had completed postgraduate training in countries ranked within the Global AI Index top-10 (54.7%).

### 3.2. Awareness, Knowledge, and Clinical Practice

Responses to the awareness, knowledge, and clinical practice domains are summarized in Table 4. Just over half of respondents were aware of AI tools or software designed for dentistry (169/300; 56.3%). Exposure was nevertheless limited: only 38.7% (116/300) had attended an AI-related lecture, workshop, or webinar, 29.0% (87/300) had read peer-reviewed AI research, and 28.7% (86/300) followed AI developments specific to dentistry. In clinical practice, only 15.7% (47/300) had ever used AI-assisted tools, although 64.0% (192/300) anticipated AI becoming integral to routine dental care. Despite limited exposure, enthusiasm was high: 94.0% (282/300) were interested in AI training, 92.0% (276/300) were open to adopting AI if proven beneficial, and 70.8% of those not already using AI (184/260) planned to do so. Confidence was more guarded, with only 45.0% (135/300) confident in their ability to adopt AI technologies.

Attitudes were broadly positive (Table 4). Most respondents were optimistic about advancements in AI for dentistry (80.7%), agreed that AI could enhance diagnostic accuracy (69.0%) and reduce errors and improve efficiency (64.7%), and believed dental schools should incorporate AI coursework (79.3%). Fewer were convinced that AI would increase patient satisfaction (57.3%) or that AI literacy conferred a competitive advantage (46.0%).

### 3.3. Beliefs Toward AI by Subgroup

The nine items forming the composite belief score showed acceptable internal consistency in the analytic sample (n = 300; KR-20 = 0.79, 95% CI: 0.75–0.82). Corrected item–total correlations ranged from 0.29 (openness to adopting AI if proven beneficial, an item with a pronounced ceiling effect: 92.0% “Yes”) to 0.60 (AI can enhance diagnostic accuracy), the mean inter-item correlation was 0.29, and removing any single item did not increase KR-20 (range with each item deleted: 0.75–0.79). The inter-item correlation matrix (Pearson correlations) was suitable for principal component analysis (KMO = 0.84; Bartlett’s test: χ^2^ = 569.9, df = 36, *p* < 0.001), and parallel analysis retained a single component (first eigenvalue = 3.38, accounting for 37.5% of the total variance; second eigenvalue = 1.07, below the corresponding 95th-percentile random-data eigenvalue of 1.23), on which all nine items loaded positively (0.39–0.74). These results support the summation of the nine items into a single score. Item-level statistics are provided in Supplementary Material Table S1.

Beliefs toward AI differed significantly by gender (Table 5; χ^2^ = 11.60, *p* = 0.003): high beliefs were more common among male (44.1%) than female (25.3%) respondents. Beliefs also differed by specialty (Table 6; χ^2^ = 18.09, *p* = 0.006), with prosthodontists the most optimistic (48.8% high beliefs) and general dentists also relatively positive (38.7% high), whereas the “others” group had the lowest proportion of high beliefs (19.3%).

In contrast, beliefs did not differ significantly by place of undergraduate education (Table 7; χ^2^ = 0.62, *p* = 0.733), years of experience (Table 8; χ^2^ = 0.70, *p* = 0.704), or work sector (Table 9; χ^2^ = 1.40, *p* = 0.844). Across all of these strata, moderate beliefs were the most common category.

### 3.4. Predictors of Belief Category (Ordinal Regression)

Results of the ordinal logistic regression are shown in Table 10. The proportional-odds assumption was satisfied (test of parallel lines: χ^2^ = 2.39, df = 6, *p* = 0.88), and no evidence of problematic multicollinearity was identified (all VIFs < 4). Male respondents had significantly greater odds of reporting higher belief categories than females (OR = 2.09, 95% CI: 1.26–3.45, *p* = 0.004). Likewise, general dentists (OR = 2.52, 95% CI: 1.38–4.61, *p* = 0.003) and prosthodontists (OR = 3.06, 95% CI: 1.48–6.33, *p* = 0.003) had significantly greater odds of higher belief categories compared with the reference specialty group. Orthodontics, place of undergraduate and postgraduate education, years of experience, and work sector were not significant predictors. The overall model was statistically significant (likelihood ratio χ^2^ = 25.56, df = 9, *p* = 0.002).

### 3.5. Barriers and Concerns

Perceived barriers to implementing AI are shown in Table 11 (n = 292; all percentages reported for this multiple-response item are based on the 292 respondents who answered it, and multiple options could be selected). The most frequently selected barrier was a lack of training and expertise among dental professionals (71.2%), followed by technical challenges in integrating AI into existing workflows (63.4%), financial cost (56.5%), and concerns about the reliability and accuracy of AI (50.3%). Data privacy and security (40.4%), ethical concerns (32.9%), and patient acceptance (22.3%) were cited less often, and only 1.4% selected no barrier. Endorsement of the two leading barriers—lack of training and cost—did not differ significantly across work sector (training: government 68.1%, private 75.8%, university 78.3%, *p* = 0.258; cost: government 56.9%, private 51.5%, university 58.3%, *p* = 0.807) or across specialty (training *p* = 0.707; cost *p* = 0.471), indicating that these barriers were pervasive across the profession.

Regarding specific concerns, 64.0% considered the cost of AI integration a significant concern and 53.8% were concerned about ethical and legal issues such as data privacy and patient consent. Fewer respondents were concerned that AI might replace dental jobs (32.9% concerned, 45.2% not concerned, 21.9% unsure).

## 4. Discussion

To our knowledge, this study provides the first insight into the awareness, attitudes, and barriers of dental professionals in Kuwait toward AI, analyzed across demographic and career-based subgroups. Because attitudes toward AI are likely to evolve as the technology advances, our findings are contrasted with the existing international literature below. The null hypothesis was rejected, as composite belief scores differed significantly among dental professional subgroups.

Several international studies have examined which specialties are seen as most amenable to AI. An Australian survey [12] found that most participants viewed maxillofacial radiology and implantology (64.5% each) as most amenable to AI. A South Korean study [13] reported that most students (70.0%) and dentists (70.8%) believed oral and maxillofacial radiology would be the first specialty to commercialize AI, consistent with an Indian study [14] in which most participants believed AI assists in interpreting head, face, and neck pathology, particularly in oral pathology (64%) and oral radiology (71%). A further Indian study [15] found that 59.5% of students were optimistic that AI would detect radiographic changes missed by clinicians, and other surveys reported that 60–70% of participants thought AI useful for the radiographic diagnosis of caries [9,16,17,18]. Pauwels and Del Rey [10] likewise reported awareness of AI in radiology but not in dentistry generally, with 33.5% agreeing that AI would replace oral radiologists within 15 years. These findings contrast with ours: in our sample, respondents in the “other specialties” group (including periodontics, surgery, and radiology) most often fell into the moderate or low belief-score categories. Because our survey did not directly measure the reasons underlying these beliefs, potential explanations remain hypotheses: the discrepancy may reflect limited exposure to and training in these technologies in Kuwait, medico-legal caution about AI reliability in high-stakes diagnostic and surgical specialties, or infrastructural limitations in access to AI-enabled imaging tools. The heterogeneous composition of the “other specialties” group may also have contributed to this pattern, and these possibilities require confirmation in future studies.

We observed a significant gender difference in the composite belief score: male dentists were more likely than female dentists to fall into the high belief category (44.1% vs. 25.3%), and male gender remained an independent predictor of higher beliefs in the adjusted regression (OR = 2.09, 95% CI: 1.26–3.45, *p* = 0.004). Because these factors were not directly measured in our survey, potential explanations—such as differences in confidence with new technologies, differential exposure to AI resources and training, or broader sociocultural factors—should be regarded as hypotheses; consistent with the exposure hypothesis, prior work has shown that dentists who attend conferences and online forums tend to be more aware of AI applications [19]. Our findings align with an Australian study [12] in which more women viewed AI as a clinical aid and preferred their own judgment in cases of discrepancy, whereas men were more inclined to use AI applications when available. A South Korean study [13], however, found that differences by age, sex, and residence were not statistically significant. Further research into gender-specific barriers and motivators would support equitable AI integration.

Regarding specialty, an Australian study [12] reported that specialists held more optimistic beliefs than general practitioners (54.5% vs. 16.2%; *p* < 0.01), attributed to greater exposure within their field. In contrast, our regression identified general dentistry (OR = 2.52, 95% CI: 1.38–4.61, *p* = 0.003) and prosthodontics (OR = 3.06, 95% CI: 1.48–6.33, *p* = 0.003) as the only significant specialty predictors of a higher belief category. A possible, though untested, explanation is that general dentists encounter a broad range of procedures and may therefore be more open to technological assistance in diagnosis, treatment planning, and administrative tasks, perhaps perceiving AI as a way to reduce cognitive load and improve efficiency; consistent with this, several studies report that the majority of trainees agree AI can improve diagnostic ability and treatment planning [9,15,18]. One hypothesis—not directly tested in our survey—is that the optimism of prosthodontists stems from their existing reliance on digital workflows (CAD/CAM systems, intraoral scanners, and 3D printing), which may make AI integration feel like a natural extension, particularly for treatment planning, occlusal analysis, and prosthesis design. A Croatian study [20] similarly found prosthodontics to be the leading area of actual AI application (13.0%), with most participants believing AI applicable to dental prosthetics (72.0%) and orthodontics (67.0%).

The limited representation of some specialties in our sample may have reduced our ability to detect significant trends, and because the reference category combined several clinically distinct specialties, the specialty-specific associations should be interpreted cautiously and not overinterpreted; analyses using the original specialty categories were not feasible owing to small cell sizes, underscoring the need for larger, stratified studies.

Our awareness findings extend the international literature to the Kuwaiti context. Awareness of dental AI tools (56.3%) was broadly comparable to the roughly 50% reported elsewhere [6,8], but a distinctive pattern emerged: enthusiasm substantially outpaced exposure. While 94.0% of respondents were interested in AI training and 92.0% were open to adoption, only 38.7% had attended any AI-related education, 29.0% had read AI research, and 15.7% had actually used AI tools. Consistent with this gap, the most frequently reported barrier was a lack of training and expertise (71.2% of the 292 respondents answering the barrier item), ahead of technical and financial barriers—mirroring the training and resource limitations that dominate the wider literature. Importantly, endorsement of the leading barriers did not differ across work sector or specialty, indicating that limited training is a profession-wide constraint in Kuwait rather than one confined to a particular setting. Together, these findings suggest that, within this self-selected sample, the most salient reported obstacle to AI adoption appeared to be limited education and hands-on experience rather than resistance—a gap that may be addressable through targeted continuing education and curricular reform, although the cross-sectional, perception-based design of this study precludes firm conclusions about actual adoption behavior.

### Limitations

This study has several limitations. First, the sample size (300 respondents in the analytic sample) and the uneven representation of some demographic groups limit the generalizability of the results. In addition, no formal a priori sample-size calculation was performed, and the study used a convenience sampling approach; therefore, the findings should be interpreted with appropriate caution. The voluntary nature of the survey introduces possible selection bias, as respondents with a particular interest in AI may have been more likely to participate. Also, the self-reported design is subject to response bias. Moreover, the composite belief score was developed specifically for this exploratory study and was not based on a previously validated instrument. Furthermore, the categorization of belief levels was investigator-defined and should therefore be interpreted with caution. The questionnaire did not undergo formal psychometric validation before distribution: although the instrument was expert-reviewed and pilot-tested, its content validity was assessed qualitatively rather than quantified, test–retest reliability was not examined, and evidence of reliability and dimensionality is available only for the nine items of the composite belief score and was obtained post hoc in the study sample. The psychometric properties of the remaining single-item measures were not established, and the structure of the composite score requires confirmation in independent samples. The classification based on national AI rankings should also be interpreted cautiously, as participants completed their training over several decades. Current rankings may not reflect the AI landscape or educational environment that existed when their training took place.

Several variables were simplified for analysis: clinically distinct specialties were combined into a heterogeneous “others” reference group, and years of experience and place of training were dichotomized, with an attendant loss of information. The cross-sectional design precludes temporal or causal inference. Finally, given the rapid evolution of AI, repeated surveys would be valuable to track how attitudes and beliefs change over time.

## 5. Conclusions

Within its limitations, this survey indicates that dental professionals in Kuwait hold broadly positive but uneven attitudes toward AI, as summarized by the composite belief score, with general dentists and prosthodontists most receptive to its future role. AI has clear potential to enhance clinical workflows and decision-making, but challenges relating to trust and ethical and legal concerns remain. Practicing dentists would benefit from hands-on training with AI tools and from continuing dental education programs, and integrating AI into dental curricula—alongside interdisciplinary collaboration between computer science and dentistry—is needed to prepare graduates to use AI effectively and responsibly.

## Figures and Tables

**Table 1:** Questionnaire items by domain (knowledge and awareness, clinical practice, attitudes, and barriers and concerns).

Knowledge and Awareness	
8	Are you aware of any AI tools or software designed for dentists?
9	Have you ever attended any lectures, workshops, or webinars related to AI in dentistry?
10	Would you be interested in participating in training programs to increase your understanding of AI in dentistry?
11	Have you read any peer-reviewed research or studies on AI applications in dentistry?
12	Do you follow updates or advancements in AI technology specific to dentistry?
**Clinical Practice**	
13	Do you anticipate AI becoming an integral part of routine dental care in the future?
14	Have you ever used AI-assisted tools or software in your dental practice?
15	(If you answered “No” previously) Are you planning to use any AI-based software or tools in your dental practice?
16	Are you confident in your ability to adopt AI technologies for your dental practice?
17	Are you open to adopting AI tools if they prove beneficial for dental procedures?
**Attitudes Toward AI**	
18	Are you optimistic about advancements in AI for dentistry in the near future?
19	Do you believe AI can enhance diagnostic accuracy in dentistry/your specialty?
20	Do you think AI can reduce errors and improve the quality and efficiency of dental procedures?
21	Do you believe the impact of AI will increase patient satisfaction?
22	Do you believe dental schools should incorporate AI-related coursework into their education programs?
23	Do you think individuals in your specialty who are well-informed about AI have a competitive advantage?
**Barriers and Concerns**	
24	Which of the following do you consider a barrier to implementing AI in your dental practice? (multiple options)
25	Are you concerned about ethical and legal issues related to AI in dentistry, such as data privacy or patient consent?
26	Do you think the cost of AI integration is a significant concern? (e.g., training, hiring AI specialists, software and hardware)
27	Are you concerned that AI might replace certain jobs currently held by individuals in dental practice?

**Table 2:** Demographic items collected from participants.

Demographics	
1	Gender
2	Date of birth
3	Specialty
4	Undergraduate education
5	Postgraduate education
6	Years of experience
7	Current work sector

**Table 3:** Frequency and percentage of participants by demographic characteristics.

Variable	Category	n (%)
Gender	Female	182 (60.7)
	Male	118 (39.3)
Specialty	General dentistry (AGD, GP, restorative)	111 (37.0)
	Orthodontics	39 (13.0)
	Prosthodontics	41 (13.7)
	Others (periodontics, surgery, endodontics, pediatric, oral medicine)	109 (36.3)
Undergraduate country (by AI rank)	Top-10 AI Index country ^a^	101 (33.7)
	Other	199 (66.3)
Postgraduate country (by AI rank)	Top-10 AI Index country ^a^	164 (54.7)
	Other	136 (45.3)
Years of experience	<3	86 (28.7)
	3–6	44 (14.7)
	6–10	52 (17.3)
	11–15	51 (17.0)
	15–20	29 (9.7)
	20+	38 (12.7)
Experience (dichotomized)	1–6 years	130 (43.3)
	>6 years	170 (56.7)
Work sector	Government	192 (64.0)
	University/academia	62 (20.7)
	Private	35 (11.7)
	Mixed/other	11 (3.7)

Analytic sample n = 300 (respondents with a complete composite belief score). Percentages are column percentages within each variable. ^a^ Countries represented in the sample and belonging to the 2024 Global AI Index top-10 group were the USA, UK, France, and India.

**Table 4:** Responses to the knowledge and awareness, clinical practice, and attitude domains (item-level valid responses).

Item	n	Yes, n (%)	Not Sure, n (%)	No, n (%)
Knowledge and awareness				
Aware of AI tools designed for dentistry	300	169 (56.3)	—	131 (43.7)
Attended an AI lecture, workshop, or webinar	300	116 (38.7)	—	184 (61.3)
Interested in AI training programs	300	282 (94.0)	—	18 (6.0)
Read peer-reviewed AI research	300	87 (29.0)	—	213 (71.0)
Follow AI advancements in dentistry	300	86 (28.7)	—	214 (71.3)
Clinical practice				
Anticipate AI becoming integral to routine care	300	192 (64.0)	96 (32.0)	12 (4.0)
Have used AI-assisted tools in practice	300	47 (15.7)	—	253 (84.3)
Plan to use AI tools (of those not yet using)	260	184 (70.8)	—	76 (29.2)
Confident in ability to adopt AI	300	135 (45.0)	118 (39.3)	47 (15.7)
Open to adopting AI if beneficial	300	276 (92.0)	20 (6.7)	4 (1.3)
Attitudes				
Optimistic about AI advancements in dentistry	300	242 (80.7)	54 (18.0)	4 (1.3)
AI can enhance diagnostic accuracy	300	207 (69.0)	77 (25.7)	16 (5.3)
AI can reduce errors and improve efficiency	300	194 (64.7)	84 (28.0)	22 (7.3)
AI will increase patient satisfaction	300	172 (57.3)	107 (35.7)	21 (7.0)
Dental schools should incorporate AI coursework	300	238 (79.3)	45 (15.0)	17 (5.7)
AI literacy confers a competitive advantage	300	138 (46.0)	117 (39.0)	45 (15.0)

**Table 5:** Belief category by gender.

Belief Category		Male	Female	Total
Low	Count	26	57	83
	% within group	22.0%	31.3%	27.7%
Moderate	Count	40	79	119
	% within group	33.9%	43.4%	39.7%
High	Count	52	46	98
	% within group	44.1%	25.3%	32.7%
Total	Count	118	182	300

χ^2^ = 11.60, df = 2, *p* = 0.003.

**Table 6:** Belief category by specialty.

Belief Category		General	Orthodontics	Prosthodontics	Others	Total
Low	Count	29	11	5	38	83
	% within group	26.1%	28.2%	12.2%	34.9%	27.7%
Moderate	Count	39	14	16	50	119
	% within group	35.1%	35.9%	39.0%	45.9%	39.7%
High	Count	43	14	20	21	98
	% within group	38.7%	35.9%	48.8%	19.3%	32.7%
Total	Count	111	39	41	109	300

χ^2^ = 18.09, df = 6, *p* = 0.006.

**Table 7:** Belief category by place of undergraduate education.

Belief Category		Other	Countries in the Global AI Index Top 10	Total
Low	Count	56	27	83
	% within group	28.1%	26.7%	27.7%
Moderate	Count	81	38	119
	% within group	40.7%	37.6%	39.7%
High	Count	62	36	98
	% within group	31.2%	35.6%	32.7%
Total	Count	199	101	300

χ^2^ = 0.62, df = 2, *p* = 0.733.

**Table 8:** Belief category by years of experience.

Belief Category		1–6 Years	>6 Years	Total
Low	Count	35	48	83
	% within group	26.9%	28.2%	27.7%
Moderate	Count	55	64	119
	% within group	42.3%	37.6%	39.7%
High	Count	40	58	98
	% within group	30.8%	34.1%	32.7%
Total	Count	130	170	300

χ^2^ = 0.70, df = 2, *p* = 0.704.

**Table 9:** Belief category by work sector.

Belief Category		University	Private	Government	Total
Low	Count	15	8	57	80
	% within group	24.2%	22.9%	29.7%	27.7%
Moderate	Count	27	14	75	116
	% within group	43.5%	40.0%	39.1%	40.1%
High	Count	20	13	60	93
	% within group	32.3%	37.1%	31.2%	32.2%
Total	Count	62	35	192	289

χ^2^ = 1.40, df = 4, *p* = 0.844. Eleven respondents reporting mixed or other work sectors were excluded from this analysis because this small (n = 11) and internally heterogeneous group could not be meaningfully analyzed as a separate sector category.

**Table 10:** Adjusted odds ratios from ordinal logistic regression predicting higher belief category toward AI.

Predictor	β Estimate	Adjusted OR	95% CI for OR	Sig.
Male vs. Female	0.736	2.09	1.26–3.45	0.004
General Dentistry vs. Others	0.924	2.52	1.38–4.61	0.003
Orthodontics vs. Others	0.521	1.68	0.81–3.50	0.164
Prosthodontics vs. Others	1.120	3.06	1.48–6.33	0.003
Undergraduate: Global AI Index vs. Others	−0.083	0.92	0.55–1.55	0.754
Postgraduate: Global AI Index Top vs. Others	0.000	1.00	0.55–1.81	1.000
Experience (1–6 years vs. >6 years)	−0.059	0.94	0.57–1.55	0.815
University vs. Government	0.201	1.22	0.71–2.11	0.469
Private vs. Government	0.332	1.39	0.68–2.84	0.362

Ordinal logistic regression with higher belief category as the outcome. OR > 1 indicates higher odds of belonging to a higher belief category. Reference categories were female gender, other specialties, non-top-10 AI countries, >6 years of experience, and government sector. Model likelihood ratio *χ^2^* = 25.56, df = 9, *p* = 0.002.

**Table 11:** Perceived barriers to implementing AI in dental practice (multiple selections allowed; n = 292).

Barrier	n (%)
Lack of training and expertise among dental professionals	208 (71.2)
Technical challenges in integrating AI into workflows	185 (63.4)
Financial barriers (cost of software, hardware, training)	165 (56.5)
Reliability and accuracy of AI technology	147 (50.3)
Resistance of some dental care providers to adoption	131 (44.9)
Data privacy and security	118 (40.4)
Ethical concerns	96 (32.9)
Patient acceptance of AI technology	65 (22.3)
None of the above	4 (1.4)

Percentages are of the 292 respondents who answered this item; multiple options could be selected.

## Data Availability

The data presented in this study are available on request from the corresponding author.

## Supplementary Materials

The following supporting information can be downloaded at https://www.mdpi.com/article/10.3390/dj14100618/s1. Supplementary Material Table S1: Item-level internal consistency and principal component statistics for the nine items of the composite belief score.
