# Supplementary material for: Awareness, Attitudes, and Barriers Toward Artificial Intelligence in Dentistry: A Cross-Sectional Survey of Dental Professionals in Kuwait
Source: Dent J (Basel). 2026 Sep 24;14(10):618. doi: 10.3390/dj14100618 (PMC13703564; doi:10.3390/dj14100618)
Supplement: Supplementary file 1 [file dentistry-14-00618-s001.zip › dentistry-4506980-supplementary.pdf]

Supplementary Material Table S1. Item-level statistics for the nine dichotomous items forming the composite belief score: endorsement, corrected item–total correlations, Kuder–Richardson 20 (KR-20) with each item deleted, and loadings on the first principal component (analytic sample, n = 300).

Item	Questionnaire item (scored 1 = Yes; 0 = No or Not sure)	Yes, %	Corrected item–total r	KR-20 if item deleted	First-component loading
13	Do you anticipate AI becoming an integral part of routine dental care in the future?	64.0	0.52	0.76	0.66
16	Are you confident in your ability to adopt AI technologies for your dental practice?	45.0	0.38	0.78	0.50
17	Are you open to adopting AI tools if they prove beneficial for dental procedures?	92.0	0.29	0.79	0.39
18	Are you optimistic about advancements in AI for dentistry in the near future?	80.7	0.51	0.76	0.65
19	Do you believe AI can enhance diagnostic accuracy in dentistry/your specialty?	69.0	0.60	0.75	0.74
20	Do you think AI can reduce errors and improve the quality and efficiency of dental procedures?	64.7	0.56	0.75	0.70
21	Do you believe the impact of AI will increase patient satisfaction?	57.3	0.49	0.76	0.63
22	Do you believe dental schools should incorporate AI-related coursework into their education programs?	79.3	0.45	0.77	0.58
23	Do you think individuals in your specialty who are well-informed about AI have a competitive advantage?	46.0	0.45	0.77	0.58

Notes. Item numbers refer to the questionnaire in Supplementary Material Table S1. Responses were scored 1 for "Yes" and 0 for "No" or "Not sure"; the composite belief score is the sum of the nine items (range 0–9; mean 5.98, SD 2.45). Overall internal consistency: KR-20 = 0.79 (95% confidence interval 0.75–0.82, Feldt method); mean inter-item correlation = 0.29 (range 0.09–0.56). Sampling adequacy: Kaiser–Meyer–Olkin measure = 0.84; Bartlett's test of sphericity $\chi^2 = 569.9$, df = 36, $p < 0.001$. Principal component analysis of the Pearson inter-item correlation matrix yielded eigenvalues of 3.38, 1.07, 0.89, 0.79, 0.71, 0.67, 0.55, 0.53 and 0.41; parallel analysis (1000 random datasets of the same dimensions, 95th percentile) retained one component (random-data thresholds for the first two eigenvalues: 1.35 and 1.23), which accounted for 37.5% of the total variance. Loadings are unrotated loadings on this first component. Corrected item–total correlations are correlations between each item and the sum of the remaining eight items.
